# With Gratitude From the Inaugural Editor-in-Chief of *Innovation in Aging*

**DOI:** 10.1093/geroni/igaa062

**Published:** 2020-11-29

**Authors:** Laura P Sands

**Affiliations:** Center for Gerontology, Virginia Tech, Blacksburg

What a tremendous privilege it has been to be the inaugural Editor-in-Chief of *Innovation in Aging*. I am truly grateful to all who contributed to the success of this journal. *Innovation in Aging* is the first online, fully open access scientific journal published by The Gerontological Society of America (GSA). The process of launching *Innovation in Aging* began as an idea. The idea was to fill an increasingly obvious gap in publishing manuscripts that did not fit within the scope, or page limitations, of other journals. *Innovation in Aging* is filling that gap, but also creating opportunities for publishing research on topics that have not been featured in gerontology and geriatrics journals. This need was identified well before I was appointed Editor-in-Chief and I am humbled to be a part of this effort. As I leave this post, I would like to take a moment to describe novel features of *Innovation in Aging*, the people who contributed to its successful launch, and my hope for the future of this journal.

## Innovation in Aging: A Toolbox for Success

First, there needed to be an additional home for the many worthy manuscripts submitted to GSA journals. Unlike print journals, *Innovation in Aging* does not have page-space limitations. Second, there was a need to develop a journal that could bring scientific perspectives and techniques that are outside the scope of existing journals. *Innovation in Aging* covers a wide range of topics relevant to older adults, from the adoption of technology to the impact of race and ethnicity on health and well-being. Third, many agencies are requiring that funded research findings be published in open-access journals. With the advice and direction from GSA and Oxford University Press (OUP) leaders, we planned the launch of *Innovation in Aging* in 2016 and began accepting submissions in January 2017.

Although the open-access model of publishing is new to GSA, *Innovation in Aging* is one of more than 1,000 fully open-access journals published by scientific societies. Open access increases readership, not just among scientists, but also with others interested in the journal’s content. Since January 2017, journal readership, submissions, and citations have increased steadily. After rigorous reviews, *Innovation in Aging* received recognition for its excellent scientific quality by being indexed in PubMed, the Emerging Sources Citation Index in Web of Science, the Directory of Open Access Journals, and SCOPUS, all of which increase the discoverability of its articles. In 4 years, *Innovation in Aging* has published 162 articles, including 3 special issues and 1 special section. *Innovation in Aging* also has gained global authorship and readership.

*Innovation in Aging* is an interdisciplinary journal. It publishes studies that explore the complex relationships between biological, physical, social, psychological, and environmental influences on the health, well-being, and productivity of older adults. We are particularly interested in studies that are the product of convergence science, an approach that is based on the collaborative contributions of team members with diverse expertise (1). For example, Dr. Nancy Morrow-Howell’s article describes how social scientists and engineers collaborated to use System Dynamics modeling to describe the complex interactions between the many psychosocial drivers of older adults’ productivity (2). Our commitment to publishing studies that bring novel approaches to solving complex issues in aging is evidenced in 14 additional studies that describe the role of technology in older adults’ lives (3–16).

*Innovation in Aging* adopted 2 features to speed the translational impact of science on aging. First, we publish Invited Articles that provide expert evaluation of the state of current science on a topic and envision directions for future discovery. The first article published in *Innovation in Aging* was in fact an Invited Article from Dr. Ken Ferraro titled, “Diverse Aging and Health Inequality by Race and Ethnicity” (17). Thus far we have published 25 Invited Articles from notable gerontologists covering a diverse set of topics including advance care planning, divorces, disability, friendships, depression, physical activity, longevity, and more. These articles provide a launching point for readers interested in developing or refining their knowledge of a topic. They also provide directions for future research which aligns with GSA’s focus on mentoring. Second, we emphasize the translational significance of study findings in a blue box just below the abstract. The translational significance describes the potential of study findings to solve a problem and speed solutions to the problem. This tool also encourages a broader readership to discover how science on aging is addressing the common challenges experienced by older adults and their care providers.

## The Journal’s Success = The People Involved. So, Thanks!

This journal would not be possible without the generous contribution of experts, especially in our special issues.

We thank Dr. Wendy Rogers, an *Innovation in Aging* Advisory Board member, who was instrumental in developing our capacity to attract and provide expert reviews of studies on technology and aging. We sincerely appreciate the leadership of Drs. Jacqueline Angel and Marc Garcia on the 2019 special section on Latino Aging and Health (Volume 3, Issue 2). We are indebted to Dr. Jill Suitor, Deputy Editor-in-Chief of *Innovation in Aging*, who expertly led the special issue on Translational Research on Caregiving (Volume 3, Issue 3). We are grateful to Dr. Vicki Freedman and Dr. Steven Albert who brought the Public Health lens to healthy aging in the Aging and Public special issue (Volume 4, Issue 1). The development of this special issue preceded the coronavirus disease 2019 pandemic which alerted the public to the critical need for public health interventions to protect older adults’ health and survival. We are grateful to Dr. Robert Taylor, who brought invaluable insight to one of the most pressing issues of our time. He was the guest Associate Editor of the special issue Race and Mental Health Among Older Adults (Volume 4, Issue 5, 2020). The development of this issue began several months prior to the height of the George Floyd protests and the Black Lives Matter demonstrations in the summer of 2020.

In addition, GSA leadership including James Appleby, Judie Lieu, Megan McCutcheon, and Managing Editors Karen Jung and Kathy Jackson provided needed guidance and support throughout the process of launching and managing the journal. David Crotty, Sara McNamara, and the staff at OUP gently educated me about the process and policies of scientific publishing. I am exceptionally grateful for the wisdom of the Advisory Board of *Innovation in Aging* who guided the development of journal policies. Deputy Editors-in-Chief Steven Albert and Jill Suitor met with me and Karen Jung weekly to plan the effective implementation of those policies. The Associate Editors have been the true heroes of the journal, making sure that submissions received quick decisions (our average time to the first decision is 25 days), and providing expert reviews of manuscripts. I read each of their decisions. I am proud to say that they were very fair and gracious in their decisions. Our Editorial Board members provided expert reviews of manuscripts and contributed excellent suggestions for improving the journal policies and scope. The names of each of our board members are listed on the journal website at https://academic.oup.com/innovateage/pages/Editorial_Board.

To reviewers of *Innovation in Aging*, your generosity of time ensured that *Innovation in Aging* met its goal of publishing innovative, conceptually sound, methodologically rigorous research on aging and the life course that has a high potential for translating scientific knowledge to improve older adults’ health, functioning, and well-being. I also want to recognize all those who submitted articles to *Innovation in Aging*. I read every submitted article and I am grateful for your dedication to the science of aging.

## Looking Toward the Future

I am looking forward to witnessing the continuing evolution and increasing impact of *Innovation in Aging* under the direction of Steven Albert, the next Editor-in-Chief. In the coming years, I expect the journal to become the home for novel, interdisciplinary approaches to the challenges in aging. I also expect the journal will increase its broad readership, including those who provide direct care or products for older adults. I also look forward to the development of online publishing features such as embedded videos and simulations in articles. New journals open opportunities; *Innovation in Aging* will continue to open opportunities for publishing and accessing novel science on aging and the life course.
